# P03-003 - Sacroilitis with propionibacterium acnes

**DOI:** 10.1186/1546-0096-11-S1-A198

**Published:** 2013-11-08

**Authors:** C Galeotti, I Koné-Paut

**Affiliations:** 1 Pediatric Rheumatology, CHU BICÊTRE, Le Kremlin Bicêtre, France

## Introduction

The syndrome of synovitis, acne, pustulosis, hyperostosis and osteitis (SAPHO) is a rare autoinflammatory syndrome, affecting essentially the adults. Osteitis with Propionibacterium Acnes reported in the literature is generally connected to the presence of a foreign body or with the SAPHO syndrome. Chronic recurrent multifocal osteomyelitis (CRMO) is a disease related to the SAPHO syndrome, affecting the children. There is essentially an isolated bone inflammation. We report the case of a 16 year-old girl, affected with a sacroiliitis for 5 years.

## Case report

This girl presented chronic pain at left sacroiliac for 5 years, with regional amyotrophy and limping. The scanner showed a left condensed sacral fine, with multiple geodes. The right sacroiliac was normal. In the hypothesis of an infection of atypical germs, a left sacroiliac biopsy was realized. There was no inflammatory infiltrate, in particular no epithelioid granuloma. But Propionibacterium acnes has been recovered from bone biopsy samples. The whole body MRI showed an aspect of sclerosis of the left sacral wing iliac joint, and the absence of lesion of the other side and the whole body. The patient was treated with NSAID then clindamycin in the hypothesis of a SAPHO syndrome. We observed a decrease of pain but the stability of the lesions after 4-months treatment with antibiotic.

## Discussion

The association sacroiliitis and P.Acnes thus directs us to a SAPHO syndrome, exceptionally reported in children; furthermore, our patient has an isolated osteitis lesion, without cutaneous lesion. Assman et al treated in 2009 30 patients having a SAPHO syndrome by azithromycin, doxycycline or clindamycin [1]. He has found P.Acnes by 14 patients who had a bone biopsy. They received a 16-week treatment with antibiotics. For the period of application, the antibiotic therapy seems to have controlled the disease. After antibiotic discontinuation, however, disease relapse was observed. Our patient could have a CRMO, which is related to the SAPHO. There is no case of CRMO associated with P.Acnes reported in the literature. On the other hand, Schilling et al treated 13 teenagers having a CRMO by azithromycin, without infectious documentation [2]. Seven children had a very fast decrease of the pain and an improvement of mobility. Other diagnostic hypothesis at these patient would be a spondylarthropathy, but the fact that she has an unilateral sacroiliitis with P.Acnes and that she has no HLA B27 antigen are not in favour. The bone biopsy finding P.Acnes guided us to a cause of ostiitis and allowed us to propose a treatment which seems to be suspensive.

## Competing interests

None Declared.

## References

[B1] Assman G Kueck O Kirchhoff T Rosenthal H Voswinkel J Pfreundschuh M Zeidler H Wagner AD Efficacy of antibiotic therapy for SAPHO syndrome is lost after its discontinuation: an interventional study Arthritis Res Ther 2009 11 5 R140 19772564 10.1186/ar2812 PMC2787295

[B2] Schilling F Wagner AD Azithromycin: an anti-inflammatory effect in chronic recurrent multifocal osteomyelitis? A preliminary report Z Rheumatol 2000 11 5 352 3 11142932 10.1007/s003930070059

